# Fracture Behavior and Integrity of Different Direct Restorative Materials to Restore Noncarious Cervical Lesions

**DOI:** 10.3390/polym13234170

**Published:** 2021-11-29

**Authors:** Emese Battancs, Márk Fráter, Tekla Sáry, Emese Gál, Gábor Braunitzer, Balázs Szabó P., Sufyan Garoushi

**Affiliations:** 1 Department of Operative and Esthetic Dentistry, Faculty of Dentistry, University of Szeged, H-6720 Szeged, Hungary; battancs.emese@gmail.com (E.B.); teklasary@gmail.com (T.S.); galmesi00@gmail.com (E.G.); 2 dicomLAB Dental Ltd., H-6726 Szeged, Hungary; braunitzergabor@gmail.com; 3 Department of Food Engineering, Faculty of Engineering, University of Szeged, H-6725 Szeged, Hungary; szpb@mk.u-szeged.hu; 4 Department of Biomaterials Science and Turku Clinical Biomaterials Center—TCBC, Institute of Dentistry, University of Turku, FI-20520 Turku, Finland; sufgar@utu.fi

**Keywords:** noncarious cervical lesion, flowable composite, glass-ionomer cement, direct restoration, fatigue resistance, microleakage, resin composite, dentistry

## Abstract

The purpose of this study was to analyze the fracture resistance and marginal leakage of noncarious cervical lesion (NCCL) restorations made of different restorative materials. Eighty upper premolars were randomly divided into four groups (*n* = 20/group). Standardized NCCL cavity preparations were performed on the buccal surface of the teeth and then restored with four different materials. Group 1: Packable resin composite (PC); Group 2: Highly flowable resin composite (HF); Group 3: Low flowable resin composite (LF); Group 4: Resin modified glass ionomer cement (RMGIC). After restorations were completed, cyclic and static fracture behavior was evaluated using a loading testing machine. Extra restored teeth were sectioned and then stained (*n* = 5/group). The specimens were viewed under a stereo microscope and the percentage of microgaps at the tooth–restoration interface was calculated. All restored teeth survived after fatigue loading. There was no statistically significant (*p* > 0.05) difference between the tested restorations after the static loading test. NCCLs restored with highly filled flowable composite showed the least microleakage among the tested groups (*p* < 0.05). The investigated restorative materials are acceptable for NCCL restorations in terms of fracture resistance and microleakage.

## 1. Introduction

A noncarious cervical lesion (NCCL) is defined as the loss of tooth structure at the cementoenamel junction (CEJ) that is not related to bacteria [1,2]. The etiology of NCCLs is considered to be multifactorial, with the proposed predisposing factors being stress (abfraction), mechanical wear (from toothbrush/dentifrice abrasion) and biocorrosion (chemical degradation) [3,4]. According to the current literature, the worldwide prevalence of NCCLs is 46.7% among adults [5]. It is important to note that the prevalence and the severity of NCCLs shows an increase with age [5,6]. Since the amount and direction of loading also appears to play an important role in the development of NCCLs [3,7], a stressful lifestyle and its intraoral sequelae, such as bruxism and temporomandibular disorders, further worsen the NCCL issue. Given that a stressful life and increased life expectancy both characterize modern western societies, the prevalence of NCCLs is expected to rise considerably in the future [8]. As noted by many, successfully restoring NCCLs remains a challenge [6,7,9,10].

Choosing the correct restorative material is of high importance in the case of restoring NCCLs. For such restorations, resin composite materials have been suggested and are routinely used [11]. However, the dentin in these lesions presents a high degree of sclerosis, with partial or total obliteration of the dentin tubules, which is unfavorable for dentin bonding [12,13]. The problem is complicated by polymerization shrinkage, which exposes the interface between the restoration and the NCCL to stress. Finally, due to their high elastic modulus, conventional packable resin composites show low flexibility when the tooth structure is deformed under mechanical load [8,14]. Consequently, the usage of flowable resin composites has been proposed for restoring NCCLs, because their elastic modulus is considerably lower, which may allow both a higher level of flexibility during function and reduced levels of stress related to polymerization shrinkage [14,15].

As an alternative to resin composite materials, modern glass ionomer materials could also be used in the case of NCCLs, since these materials are especially suitable for bonding with sclerotic dentin [13].

The question arises as to which of the abovementioned materials would be best to restore an NCCL in terms of fracture resistance. The marginal leakage of these restorations is also an open question. Therefore, the purpose of this study was to analyze the fracture resistance and marginal leakage of NCCL restorations made of different restorative materials.

The null hypothesis is that (1) NCCLs restored with various filling materials would have no difference in mechanical resistance, and (2) in microleakage values.

## 2. Materials and Methods

The study was approved by the Ethics Committee of the University of Szeged, Szeged, Hungary. The study design conformed to the Declaration of Helsinki in all respects. Eighty upper premolar teeth, extracted for periodontal or orthodontic causes, were used. The required sample size was calculated in G*Power 3.1.9.7 (Universität Düsseldorf, Düsseldorf, Germany), for four groups and an effect size of *f* = 0.4, for ANOVA, and a statistical power of 0.8. The required total sample size turned out to be *n* = 76. The freshly extracted premolars were placed into 5.25% NaOCl for 5 min and then hand scalers were used to remove soft tissues [16,17,18]. All teeth were stored in 0.9% saline solution at room temperature and were used within 2 months.

The inclusion criteria were as follows: visual absence of caries or root cracks, absence of previous endodontic treatment, posts, crowns, or resorptions. The specimens were also selected according to their coronal and radicular dimensions, measured vertically, mesio-distally, and bucco-palatally. Regarding these dimensions, the deviation limit was 10% from the group mean.

### 2.1. Cavity Preparation and Restorative Procedures

A standardized NCCL was prepared on the buccal part of all teeth using a ball diamond bur (801.016.018 FG—Brasseler USA Dental, Savannah, GA, USA) with water coolant. The artificial lesions were 2 mm high (corono-apically, ending 1 mm above and 1 mm below the cementoenamel junction (CEJ)), 4 mm wide (mesio-distally), and 2 mm deep in the midline of the lesion. The lesion dimensions were continuously controlled with the size of the bur used for preparation and with a 15 UNC periodontal probe (Hu-Friedy Mfg. Co., Chicago, IL, USA). Four different materials were used to restore the specimens (Table 1). The teeth were evenly divided into four groups (*n* = 20/group, Groups 1–4).

All specimens in Groups 1–3 received the same adhesive treatment. The enamel was selectively acid-etched with 37% phosphoric acid (Ultraetch, Ultradent, South Jordan, UT, USA) for 15 s and washed with water. After drying the cavities, adhesive treatment was carried out with G-Premio Bond (GC Europe, Leuven, Belgium) according to the manufacturer’s instructions. The adhesive was light-cured for 40 s with a 501 quartz-tungsten-halogen light-curing unit (Kerr Corp., Orange, CA, USA). The average power density of the light source, measured with a digital radiometer (Jetlite light tester, J. Morita USA Inc., Irvine, CA, USA), was 780 ± 36.8 mW/cm^2^.

**Figure 1:**
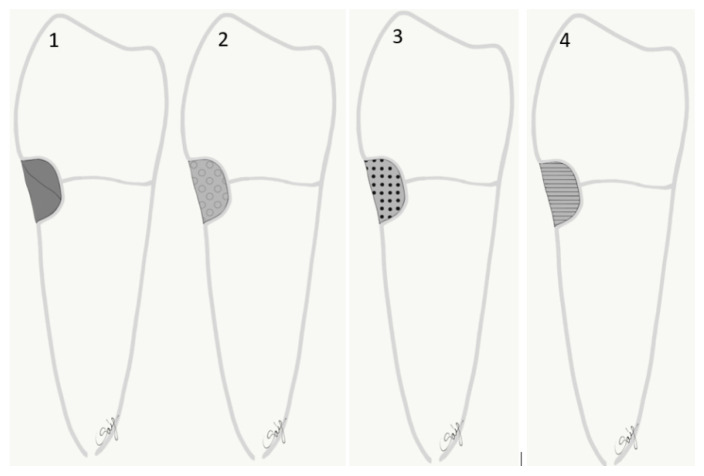
Diagram showing the test groups restored with different direct filling materials. 1: packable composite PC; 2: high flowable composite HF; 3: low flowable composite LF; 4: resin modified glass ionomer cement RMGIC.

The specimens were restored as follows (Figure 1):

Group 1: The teeth were restored with packable (PC) resin composite (Essentia Universal, GC Europe). The resin composite was applied in two consecutive oblique increments. Each layer was light-cured for 20 s.

Group 2: The teeth were restored with a less filled flowable (HF) resin composite (Essentia HiFlo, GC Europe, Leuven, Belgium) the same way as in Group 1.

Group 3: The teeth were restored with a highly filled flowable (LF) resin composite (Essentia LoFlo, GC Europe, Leuven, Belgium) the same way as in Group 1.

Group 4: The cavities were conditioned with 10% polyacrylic acid (Dentin Conditioner, GC Europe), and then washed and lightly dried. The teeth were restored with resin modified GIC (Fuji II LC, GC Europe, Leuven, Belgium) applied and light-cured according to the respective manufacturers’ instructions.

The restorations were finished with a fine granular diamond bur (FG 7406-018, Jet Diamonds, Ft. Worth, TX, USA and FG 249-F012, Horico, Berlin, Germany) under water cooling. The restored specimens were stored in a physiological saline solution (Isotonic Saline Solution 0.9% B.Braun, Melsungen, Germany) until testing.

### 2.2. Mechanical Testing

As described in our previous article [16], the root surface of the restored specimen was coated with a layer of liquid latex separating material (Rubber-Sep, Kerr, Orange, CA, USA) prior to embedding. To simulate the normal bone level, the specimens were embedded in methacrylate resin (Technovit 4004, Heraeus-Kulzer, Hanau, Germany) at 2 mm below the CEJ.

**Figure 2:**
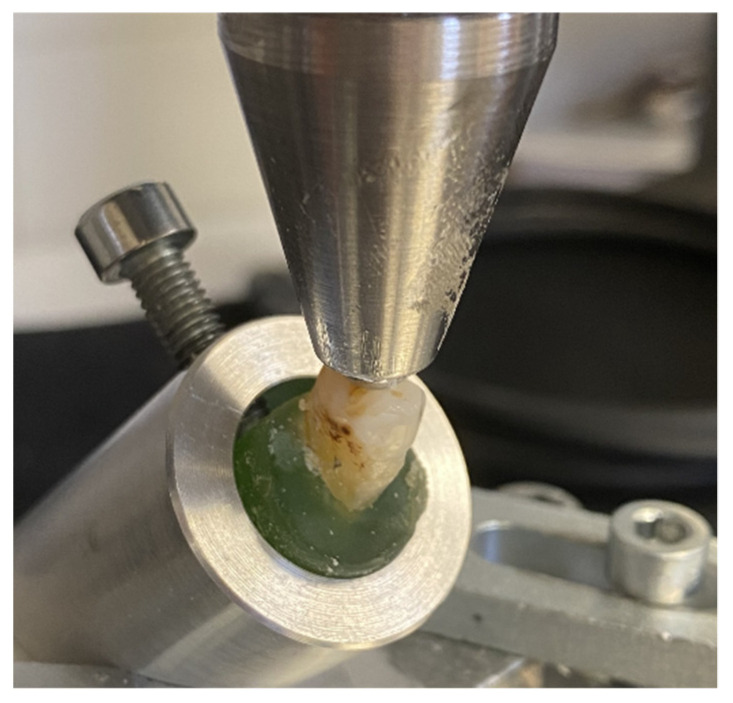
A photograph showing the test specimen and the fatigue load test setup.

Mechanical testing was carried out in two phases. In the first phase (pretesting), all restored specimens were submitted to an accelerated fatigue-testing protocol [19,20] by a hydrodynamic testing machine (Instron ElektroPlus E3000, Norwood, MA, USA) at an angle of 135 degrees to the long axis of each tooth (Figure 2).

This phase simulated normal biting forces. Cyclic isometric loading was applied with a round-shaped metallic tip to the triangular ridge of the buccal cusp. A cyclic loading of 5 Hz was applied, starting with gradually increasing static loading till 100 N in 5 s, followed by cyclic loading in 100 N steps up to 500 N, with 5000 cycles per step. The specimens were loaded until fracture occurred or up to 25,000 cycles.

In the second phase, the surviving specimens underwent static load-to-fracture testing (Lloyd R1000, Lloyd Instruments Ltd., Fareham, UK) at a crosshead speed of 0.5 mm/min. This phase simulated traumatic forces. A force vs. extension curve was dynamically plotted for each specimen. Fracture threshold, defined as the load at which the tooth–restoration complex exhibited the first fracture (detectable as peak formation on the extension curve), was recorded in Newtons (N).

### 2.3. Microleakage Analysis

**Figure 3:**
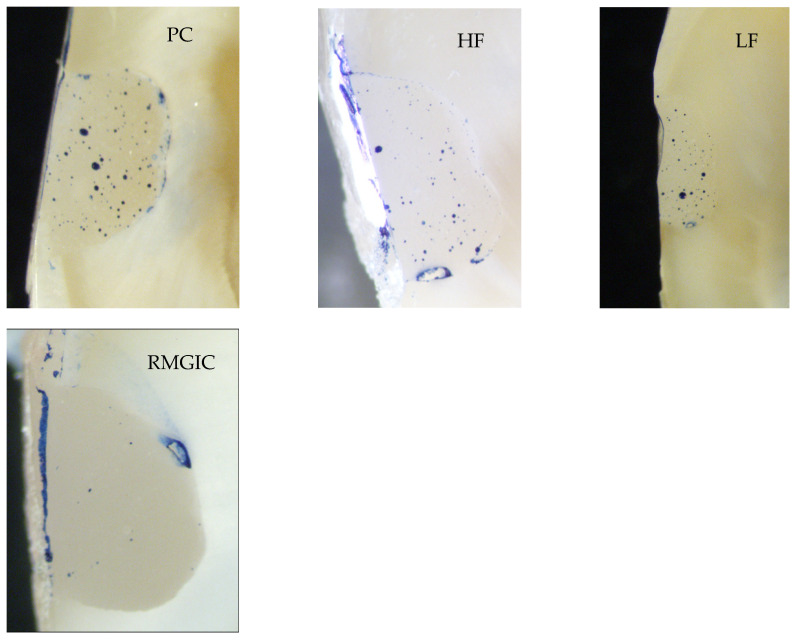
Pictures of sectioned specimens from all groups (1–4) showing microgapsat the restoration–tooth interface.

Five restored teeth per group were investigated in the microleakage analysis test. The teeth were restored and fatigued in the same way as mentioned earlier (Groups 1–4). The teeth were sectioned mid-sagitally in the buccal–lingual plane using a ceramic cutting disc operating at a speed of 100 rpm (Struers, Glasgow, Scotland) under water cooling. Following this, the sectioned teeth were further ground and polished using #4000-grit silicon carbide papers at 300 rpm under water cooling using an automatic grinding machine (Rotopol-1; Struers, Copenhagen, Denmark). The sectioned teeth were then painted with permanent marker, and polished gently for few seconds. The dye penetration along the restoration margins of each section was evaluated independently using a stereo microscope (Heerbrugg M3Z, Heerbrugg, Switzerland) at a magnification of 6.5×, and the extent of dye penetration was recorded in mm as a percentage of the total margin length (Figure 3).

### 2.4. Statistical Analysis

Statistical analysis was performed in SPSS 23.0 (IBM Corp., Somers, NY, USA). The number of survived cycles was analyzed descriptively for each group and with the Kaplan–Meier method across the groups (with the Breslow test for the pairwise analyses). The frequency of restorable and non-restorable fractures, as well as the number of survived teeth, was calculated for each group. For the comparisons between the survived samples, an ANOVA with Tukey’s HSD post hoc test was used. The normality of the data was tested by both the Kolmogorov–Smirnov and the Shapiro–Wilk tests. For both tests and all groups, the level of significance was *p* > 0.05, indicating that the data were normally distributed.

## 3. Results

**Figure 4:**
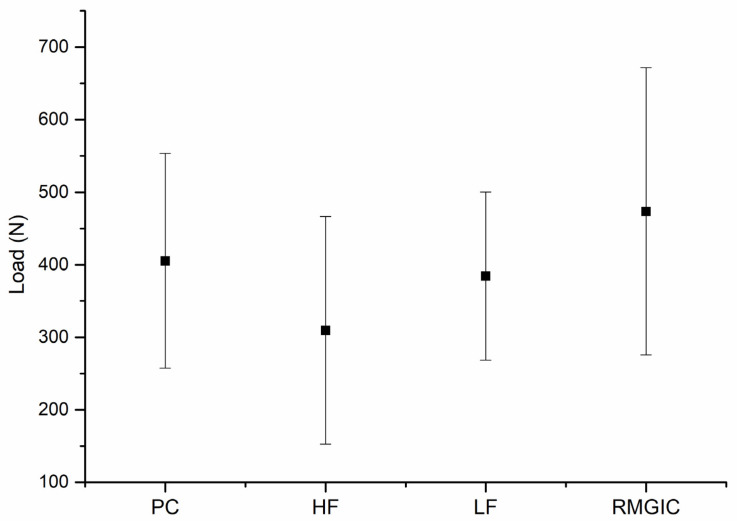
The mean values for the fracture loads (Newton) and standard deviation of the restored teeth.

Ten teeth were excluded (2–3/group) during the dynamic mechanical testing due to initial failure (fracture of the embedding material). The rest of the restored specimens survived the fatigue loading. Table 2 and Figure 4 show the descriptive fracture resistance values (mean, standard deviation, minimum, median, and maximum values) of the previously surviving specimens under static loading.

**Figure 5:**
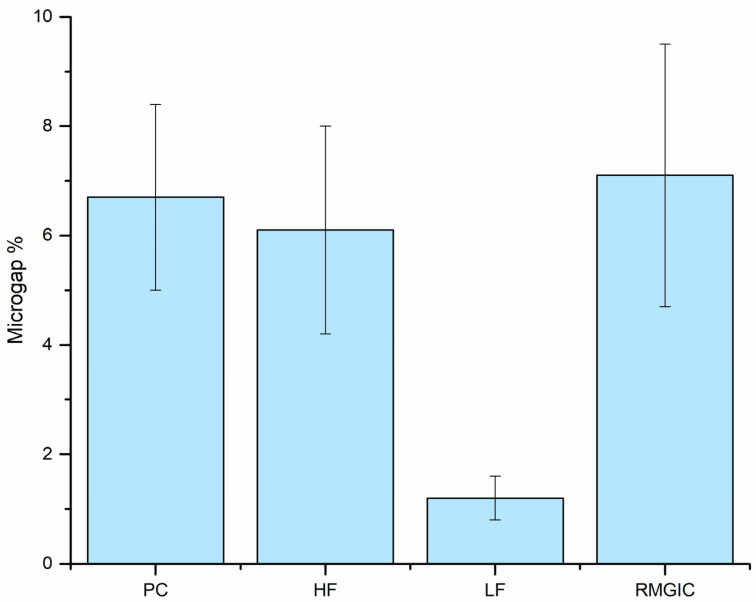
Mean percentage of microgaps and standard deviation observed in different groups from total restoration–tooth interface length after staining.

There was no statistically significant difference between the tested restorations (*p* > 0.05); thus, the first null hypothesis was accepted. Regarding the microleakage testing (Figure 5), NCCLs restored with highly filled flowable composite showed significantly (*p* < 0.05) the least microleakage among the tested groups. Therefore, the second null hypothesis was rejected. In the rest of the groups, the microleakage was rather similar (*p* > 0.05).

## 4. Discussion

This study intended to analyze the fracture behavior and microleakage of different cervical restorations used for restoring NCCLs. In our study, upper premolar teeth were used. The reason for this is that premolars are exposed to a combination of compressive and shear forces during mastication, which puts these teeth at an increased risk of cusp fracture [21]. As Tangsripongkul and colleagues point out, when a nonaxial load is applied to the upper premolars, the tensile stress concentration at the cervical area and adjacent root surface increases [2]. Furthermore, as shown by Wood et al., NCCLs manifest primarily on upper premolars [22]. The tested specimens were loaded at an angle of 45° to the long axis of the tooth, which is generally used for the mechanical testing of premolar teeth [23]. First, the restored specimens were pretested and fatigued with cyclic loading during accelerated fatigue testing [20]. It is known that cycling fatigue loading simulates the clinical situation better compared to static loading, since cyclic forces similar to normal masticatory forces are generated during the testing. The accelerated fatigue protocol was introduced as a middle ground between the classic load-to-fracture test and the more sophisticated, but also time-consuming, fatigue tests [24,25,26]. As all specimens survived the pretesting phase, load-to-fracture testing was also carried out in the entire sample. Dynamic loading is intended to simulate normal forces occurring during masticatory movements, while static loading (i.e., load-to-fracture testing) rather mimics the occurrence of traumatic forces when biting on a foreign or hard object (e.g., seed, etc.), or when suffering trauma or during bruxing or clenching. While dynamic loading is always more relevant, as the number of bruxing increases with changing lifestyle in western societies, simulating traumatic forces is becoming more and more relevant [16,27].

To our knowledge, studies that involve mechanical testing to compare different restorative materials for treating NCCLs are rare. In our study, there was no statistically significant difference between the fracture resistance of the tested groups. Therefore, the first null hypothesis was accepted. It appears that, considering fracture resistance, NCCLs may be treated adequately with any of the tested materials. This is contrary to the findings of Ichim et al., who found that none of the proposed materials were ideal for restoring NCCLs [9]. At the same time, our findings are in line with clinical data on the longevity of direct restorations used in NCCLs [28,29]. When analyzing the failed specimen, no association was found between the applied restoration and the location of the fracture.

It is well known that the retention of NCCL restorations relies primarily on adhesion, given the lack of inherent macro-mechanical retention in these lesions [8,14,15]. In general, the marginal adaptation of direct composite restorations can be influenced by the type of the applied adhesive system and factors related to the development of stress during polymerization [13,30,31]. Polymerization stress is affected by multiple factors, such as elastic modulus [32], volume factor and cavity geometry [33], the restorative technique, and the light-curing protocol [34,35]. Polymerization shrinkage-related stress develops at the tooth–restoration interface, potentially resulting in subsequent marginal gaps, microleakage, and even micro-cracking, leading to degradation and marginal staining in time [36]. Thus, microleakage was also examined in our study. Restorations made with a low flowability flowable resin composite showed the lowest amount of marginal leakage. Thus, the second null hypothesis was rejected. Furthermore, in terms of microleakage, there was no difference between the RMGIC and packable resin composite restorations. This is contrary to the findings of Anhesini et al., who found that, after being loaded, NCCLs restored with packable resin composite were characterized by higher microleakage than those restored with RMGIC [37]. However, their loading protocol differed from ours. Our in vitro results corroborate the results of those clinical studies that failed to show a considerable difference between the success of GIC and packable resin composite materials when used to restore NCCLs [38,39]. Obviously, GIC/RMGIC materials allow poorer adhesion than modern adhesives, but this does not seem to affect the longevity of cervical restorations made with GIC/RMGIC. In fact, in their systematic review and meta-analysis, Boing and colleagues demonstrated that NCCL restorations made of GIC were superior to composite restorations in terms of survival [40].

As mentioned before, the use of low flowability flowable resin composite (Essentia LoFlo) was associated with superior microleakage values in this study. According to many, the elastic modulus of restorative materials shows a positive correlation with the distribution of stress in the tooth [41,42,43]. While an increase in filler content does lead to decreased volumetric contraction [44], this also results in rigid materials with high elastic modulus, which means higher tension for the same amount of shrinkage [30]. The results may suggest that this particular type of flowable resin composite exhibits an optimal balance of these characteristics for the given indication, but the filling protocol might have played a role as well. The authors would like to stress that all resin composite materials used in this study were applied according to an oblique layering protocol. It is known that incremental filling in oblique layers, with increments of less than 2 mm, reduces polymerization stress through reduced cavity configuration factor (C-factor) and resin composite thickness [15].

Nevertheless, our results should be interpreted in a global picture as new alternatives are rising to aid the battle against marginal degradation and secondary caries. For example, recent research has shown the deposition of hydroxyapatite on polymeric resin composite [45]. This could represent additional help in the prevention of secondary caries on the margins of restorations. In the future, this feature could also be tested with the materials reported in the present investigation.

In this study, the clinically most frequently used materials (packable resin composite, flowable resin composite, and RMGIC) for restoring NCCLs were tested. The tested materials are good representatives of their material groups; however, as individual differences are present in the composition between different packable or flowable composites, the results may be altered when testing other materials with different mechanical or polymerization features. Furthermore, bulk fill composites and modern, non-resin-containing glass ionomer restorative materials (e.g., Equia Forte) can also be used for this indication. As some bulk-fill composites show unique features in polymerization-related stress, this can certainly influence the marginal leakage and microgap formation of their restorations, thus holding potential if tested in such indications.

In our study, the periodontal ligaments were simulated with a thin layer of latex separating material (Rubber-Sep). As periodontal ligaments establish a functional connection between the alveolar bone and the cementum covering the root surface, they play a crucial role in force transmission, enabling some minor movement of the teeth in the alveolus. In their absence, the model would simulate a clinically rare, so-called ankylotic condition, which is more relevant when examining implants. The usage of a latex coating on the root surface is considered a strength of our study. Furthermore, we used both cyclic and static loading, and also microleakage testing, which we consider a strength.

On the other hand, cyclic loading was not performed in a fluid chamber, and this weakens the comparability of our results to those of in vivo studies where saliva is always present during the loading cycles. This is a limitation to be addressed in our upcoming studies. Furthermore, modern glass ionomer hybrid materials should also be tested for this specific indication.

## 5. Conclusions

Based on the findings, we concluded that the investigated restorative materials (packable/flowable resin composite and RMGIC) withstood accelerated dynamic loading conditions when used for restoring NCCLs. Furthermore, the different restorative materials did not differ in their fracture resistance values. In terms of microleakage, all tested materials were deemed acceptable for NCCL restorations. As the analyzed materials are the most frequently used materials for restoring NCCLs in clinical practice, it seems safe to state that, when being able to apply according to the manufacturer’s instructions and best practice (isolation issues, etc.), all proposed materials can be suitable in this frequent and relevant clinical condition.

## Figures and Tables

**Table 1:** List of the materials used during the restorative procedures in this study.

Materials Used in This Study		
Material	Commercial Name	Composition
Packable resin composite	GC Essentia Universal Composite	urethane dimethacrylate (UDMA), bismethacrylate (BisEMA), dimethylmethacrylate, isopropylidenediphenol, methylpropenoic acid, benzotriazolcresol. Prepolymerized silica and ytterbium trifluoride, barium glass 81 weight%
Flowable resin composite		
	GC Essentia LoFlo	UDMA, dimethylmethacrylate, benzotriazolcresol, fomardehyde polymers, diphenylphosphine oxide. Barium glass 69 weight%. Differences in the fillers size
	GC Essentia HiFlo	
RMGIC	GC Fuji II LC in caps	2-hydroxyethyl methacrylate, polyacrylic acid, water. 58 weight% Fluoro-aluminumsilicate
Adhesive system	G-Premio Bond	methacryloyloxydecyl dihydrogen phosphate, methacryloxyethyl trimellitate, methacryloyloxyalkyl thiophosphate methylmethacrylate, butylated hydroxytoluene, acetone, dimethacrylateresins, initiators, water
Dentin Conditioner	GC Dentin Conditioner	10% polyacrylic acid
Etching gel	Ultradent-Ultra-Etch	orthophosphoric acid 35%

**Table 2:** Descriptive statistics of fracture load (in Newtons).

Group	N	Mean	SD	Median	Minimum	Maximum
PC	18	405.44	148.784	365.50	238	844
HF	15	309.47	157.855	310.00	89	610
LF	14	384.29	116.975	388.00	187	578
RMGIC	18	473.50	198.540	418.50	202	903

## Data Availability

Data are contained within the article.

## References

[1] Sawlani K., Lawson N.C., Burgess J.O., Lemons J.E., Kinderknecht K.E., Givan D.A., Ramp L. (2016). Factors influencing the progression of noncarious cervical lesions: A 5-year prospective clinical evaluation. J. Prosthet. Dent..

[2] Tangsripongkul P., Jearanaiphaisarn T. (2020). Resin Composite Core and Fiber Post Improved the Fracture Parameters of Endodontically Treated Maxillary Premolars with Wedge-shaped Cervical Lesions. J. Endod..

[3] Zeola L., Pereira F.A., Machado A.C., Reis B.R., Kaidonis J., Xie Z., Townsend G.C., Ranjitkar S., Soares P.V. (2016). Effects of non-carious cervical lesion size, occlusal loading and restoration on biomechanical behaviour of premolar teeth. Aust. Dent. J..

[4] Grippo J.O., Simring M., Coleman T.A. (2011). Abfraction, Abrasion, Biocorrosion, and the Enigma of Noncarious Cervical Lesions: A 20-Year Perspective. J. Esthet. Restor. Dent..

[5] Teixeira D.N.R., Thomas R.Z., Soares P.V., Cune M.S., Gresnigt M.M., Slot D.E. (2020). Prevalence of noncarious cervical lesions among adults: A systematic review. J. Dent..

[6] Schroeder M., Reis A., Luque-Martinez I., Loguercio A.D., Masterson D., Maia L.C. (2015). Effect of enamel bevel on retention of cervical composite resin restorations: A systematic review and meta-analysis. J. Dent..

[7] Peumans M., Politano G., Van Meerbeek B. (2020). Treatment of noncarious cervical lesions: When, why, and how. Int. J. Esthet. Dent..

[8] May S., Cieplik F., Hiller K.-A., Buchalla W., Federlin M., Schmalz G. (2017). Flowable composites for restoration of non-carious cervical lesions: Three-year results. Dent. Mater..

[9] Ichim I.P., Schmidlin P.R., Lic Q., Kieser J.A., Swain M. (2007). Restoration of non-carious cervical lesions: Part II. Restorative material selection to minimise fracture. Dent. Mater..

[10] Perez C.d.R., Gonzalez M.R., Prado N.A., de Miranda M.S., Macêdo M.d.A., Fernandes B.M. (2012). Restoration of noncarious cervical lesions: When, why, and how. Int. J. Dent..

[11] Schwendicke F., Müller A., Seifert T., Jeggle-Engbert L.-M., Paris S., Göstemeyer G. (2021). Glass hybrid versus composite for non-carious cervical lesions: Survival, restoration quality and costs in randomized controlled trial after 3 years. J. Dent..

[12] Luque-Martinez I., Mena-Serrano A., Muñoz M.A., Hass V., Reis A., Loguercio A. (2013). Effect of Bur Roughness on Bond to Sclerotic Dentin With Self-etch Adhesive Systems. Oper. Dent..

[13] Loguercio A.D., Luque-Martinez I.V., Fuentes S., Reis A., Muñoz M.A. (2017). Effect of dentin roughness on the adhesive performance in non-carious cervical lesions: A double-blind randomized clinical trial. J. Dent..

[14] Pecie R., Krejci I., García-Godoy F., Bortolotto T. (2011). Noncarious cervical lesions (NCCL)—A clinical concept based on the literature review. Part 2: Restoration. Am. J. Dent..

[15] Kubo S., Yokota H., Yokota H., Hayashi Y. (2010). Three-year clinical evaluation of a flowable and a hybrid resin composite in non-carious cervical lesions. J. Dent..

[16] Fráter M., Forster A., Keresztúri M., Braunitzer G., Nagy K. (2014). In vitro fracture resistance of molar teeth restored with a short fibre-reinforced composite material. J. Dent..

[17] Demiryürek E.Ö., Külünk Ş., Saraç D., Yüksel G., Bulucu B. (2009). Effect of different surface treatments on the push-out bond strength of fiber post to root canal dentin. Oral Surg. Oral Med. Oral Pathol. Oral Radiol. Endodontol..

[18] Asnaashari M., Kooshki N., Salehi M.M., Azari-Marhabi S., Moghadassi H.A. (2020). Comparison of Antibacterial Effects of Photodynamic Therapy and an Irrigation Activation System on Root Canals Infected With Enterococcus faecalis: An In Vitro Study. J. Lasers Med. Sci..

[19] Fráter M., Sáry T., Braunitzer G., Szabó P.B., Lassila L., Vallittu P.K., Garoushi S. (2021). Fatigue failure of anterior teeth without ferrule restored with individualized fiber-reinforced post-core foundations. J. Mech. Behav. Biomed. Mater..

[20] Fráter M., Sáry T., Vincze-Bandi E., Volom A., Braunitzer G., P. B.S., Garoushi S., Forster A. (2021). Fracture Behavior of Short Fiber-Reinforced Direct Restorations in Large MOD Cavities. Polymer.

[21] Robbins J. (2002). Restoration of the endodontically treated tooth. Dent. Clin. N. Am..

[22] Wood I., Jawad Z., Paisley C., Brunton P. (2008). Non-carious cervical tooth surface loss: A literature review. J. Dent..

[23] Wandscher V., Bergoli C.D., Limberger I., Ardenghi T., Valandro L. (2014). Preliminary Results of the Survival and Fracture Load of Roots Restored With Intracanal Posts: Weakened vs. Nonweakened Roots. Oper. Dent..

[24] Magne P., Lazari P., Carvalho M., Johnson T., Cury A.D.B. (2017). Ferrule-Effect Dominates Over Use of a Fiber Post When Restoring Endodontically Treated Incisors: An In Vitro Study. Oper. Dent..

[25] Lazari P., Carvalho M.A., Cury A.A.D.B., Magne P. (2018). Survival of extensively damaged endodontically treated incisors restored with different types of posts-and-core foundation restoration material. J. Prosthet. Dent..

[26] Magne P., Carvalho A., Bruzi G., Anderson R., Maia H., Giannini M. (2014). Influence of No-Ferrule and No-Post Buildup Design on the Fatigue Resistance of Endodontically Treated Molars Restored With Resin Nanoceramic CAD/CAM Crowns. Oper. Dent..

[27] Barbosa Tde S., Miyakoda L.S., Pocztaruk Rde L., Rocha C.P., Gavião M.B. (2008). Temporomandibular disorders and bruxism in childhood and adolescence: Review of the literature. Int. J. Pediatr. Otorhinolaryngol..

[28] Cieplik F., Scholz K.J., Tabenski I., May S., Hiller K.-A., Schmalz G., Buchalla W., Federlin M. (2017). Flowable composites for restoration of non-carious cervical lesions: Results after five years. Dent. Mater..

[29] van Dijken J.W., Sunnegårdh-Grönberg K., Lindberg A. (2007). Clinical long-term retention of etch-and-rinse and self-etch adhesive systems in non-carious cervical lesions: A 13 years evaluation. Dent. Mater..

[30] Fagundes T., E Barata T.J., Bresciani E., Santiago S., Franco E.B., Lauris J., Navarro M.F.L. (2014). Seven-Year Clinical Performance of Resin Composite Versus Resin-Modified Glass Ionomer Restorations in Noncarious Cervical Lesions. Oper. Dent..

[31] Correia A.M.D.O., Tribst J.P.M., Matos F.D.S., Platt J.A., Caneppele T., Borges A.L.S. (2018). Polymerization shrinkage stresses in different restorative techniques for non-carious cervical lesions. J. Dent..

[32] Braga R.R., Yamamoto T., Tyler K., Boaro L., Ferracane J., Swain M. (2012). A comparative study between crack analysis and a mechanical test for assessing the polymerization stress of restorative composites. Dent. Mater..

[33] Borges A.L.S., Borges A.B., Xavier T.A., Bottino M.C., Platt J.A. (2014). Impact of Quantity of Resin, C-factor, and Geometry on Resin Composite Polymerization Shrinkage Stress in Class V Restorations. Oper. Dent..

[34] Santos G.O., Silva A.H., Guimarães J.G., Barcellos A.D., Sampaio E.M., Silva E.M. (2007). Analysis of gap formation at tooth-composite resin interface: Effect of C-factor and light-curing protocol. J. Appl. Oral Sci..

[35] Braga R.R., Ballester R.Y., Ferracane J. (2005). Factors involved in the development of polymerization shrinkage stress in resin-composites: A systematic review. Dent. Mater..

[36] Gamarra V.S.S., Borges G.A., Burnet L.H., Spohr A.M. (2017). Marginal adaptation and microleakage of a bulk-fill composite resin photopolymerized with different techniques. Odontology.

[37] Anhesini B.H., Landmayer K., Nahsan F.P.S., Pereira J.C., Honório H.M., Francisconi-Dos-Rios L.F. (2018). Composite vs. ionomer vs. mixed restoration of wedge-shaped dental cervical lesions: Marginal quality relative to eccentric occlusal loading. J. Mech. Behav. Biomed. Mater..

[38] Vural U.K., Meral E., Ergin E., Gürgan S. (2020). Twenty-four-month clinical performance of a glass hybrid restorative in non-carious cervical lesions of patients with bruxism: A split-mouth, randomized clinical trial. Clin. Oral Investig..

[39] Bezerra I.M., Brito A.C.M., de Sousa S.A., Santiago B.M., Cavalcanti Y.W., Almeida L.D.F.D.D. (2020). Glass ionomer cements compared with composite resin in restoration of noncarious cervical lesions: A systematic review and meta-analysis. Heliyon.

[40] Boing T.F., de Geus J.L., Wambier L.M., Loguercio A.D., Reis A., Mongruel Gomes O.M. (2018). Are Glass-Ionomer Cement Restorations in Cervical Lesions More Long-Lasting than Resin-based Composite Resins? A Systematic Review and Meta-Analysis. J. Adhes. Dent..

[41] Kim R.J.-Y., Kim Y.-J., Choi N.-S., Lee I.-B. (2015). Polymerization shrinkage, modulus, and shrinkage stress related to tooth-restoration interfacial debonding in bulk-fill composites. J. Dent..

[42] Bicalho A., Valdívia A., Barreto B., Tantbirojn D., Versluis A., Soares C. (2014). Incremental Filling Technique and Composite Material—Part II: Shrinkage and Shrinkage Stresses. Oper. Dent..

[43] Soares C.J., Bicalho A.A., Tantbirojn D., Versluis A. (2013). Polymerization Shrinkage Stresses in a Premolar Restored with Different Composite Resins and Different Incremental Techniques. J. Adhes. Dent..

[44] El-Damanhoury H., Platt J.A. (2014). Polymerization Shrinkage Stress Kinetics and Related Properties of Bulk-fill Resin Composites. Oper. Dent..

[45] Butera A., Pascadopoli M., Gallo S., Lelli M., Tarterini F., Giglia F., Scribante A. (2021). SEM/EDS Evaluation of the Mineral Deposition on a Polymeric Composite Resin of a Toothpaste Containing Biomimetic Zn-Carbonate Hydroxyapatite (microRepair^®^) in Oral Environment: A Randomized Clinical Trial. Polymer.

